# Validity of a questionnaire on self-efficacy for Pap test adherence screening

**DOI:** 10.3389/fonc.2022.979799

**Published:** 2022-10-26

**Authors:** María-Teresa Urrutia, Oslando Padilla

**Affiliations:** ^1^ School of Nursing, Universidad Andres Bello, Santiago, Chile; ^2^ Department of Public Health, School of Medicine, Pontificia Universidad Católica de Chile, Santiago, Chile

**Keywords:** uterine cervical neoplasms, Papanicolaou test, self-efficacy, reproducibility of results, surveys and questionnaires. 2

## Abstract

**Introduction:**

Self-efficacy has been related to different health preventive behaviors, included adherence to the Papanicolaou test—also called Pap smear or Pap test. The aim of this study is to test construct and criterion validity and reliability of a questionnaire on self-efficacy and the Pap test in Chilean women.

**Method:**

This study was carried out on a sample of 969 women of ages from 25 to 64, who are users of the public health care system in Santiago, Chile. The validity of the Self-Efficacy Scale for the Pap Smear Screening Participation (SES-PSSP) questionnaire was done by confirmatory factor analysis, external criteria by t-test, and reliability by Cronbach’s alpha.

**Results:**

Three models were tested, obtaining a questionnaire with 20 items and 2 dimensions. The criteria validity was confirmed by adherence to the Pap test. The final questionnaire has a reliability of 0.95, measured by Cronbach´s alpha.

**Conclusion:**

A valid and reliable questionnaire to measure self-efficacy in relation to the Pap test is a relevant contribution in cervical cancer prevention, especially related to interventions focused on increasing adherence.

## Introduction

The need to explain behavior has been the motivation of many health theorists. Bandura’s Social Cognitive Theory (1) establishes self-efficacy among its main components, defined as the perception of control that can be exercised over a certain health behavior. The level of self-efficacy affects the choices people make, how much effort they invest, and how long they will persist in carrying out a certain behavior (2). The higher the level of self-efficacy, the greater the commitment to comply with a certain health behavior and the lower the perception of obstacles to carrying it out (1).

Self-efficacy has been related to different preventive health behaviors, such as screening for breast cancer (3–8), colon cancer (7, 9–11) and certain preventive practices in skin cancer (12). Regarding cervical cancer (CC), its relationship with adherence to the human papillomavirus vaccine (13, 14), adherence to the Papanicolaou (Pap) test—also called Pap smear or Pap—and to colposcopy (15), to educational interventions (16–21) and to depressive symptoms in women with the disease (22) has been studied.

The relationship between self-efficacy and adherence to Pap tests has also been studied (23–32) and found to establish that high levels of self-efficacy predict both the behavior of adhering to screening (33–41), as well as the intention (35, 42, 43). The participants’ CC and Pap test screening knowledge levels increased as their self-efficacy levels increased (44).

Given the importance of CC prevention and the relationship with self-efficacy, it is relevant to have a valid and reliable instrument in a commonly spoken and understood language that allows measurement of the self-efficacy of women in relation to adherence to CC screening. The aim of this work is to validate an instrument on self-efficacy related to Pap tests in Chilean women, written in Spanish.

## Materials and methods

The study is part of the FONDECYT #11130626 grant, “Social determinants for adherence to CC screening.” The universe of study corresponds to women from ages 25 to 64 years, belonging to the Chilean public health system—National Health Fund (FONASA)—and registered in one of the four primary health care centers of the Puente Alto commune in Santiago, Chile. The sample was selected and stratified by health centers and Pap test coverage levels. According to Pap test coverage data, four primary health care centers were randomly selected, with probabilities proportional to their size, one from each group: with the highest coverage, medium-high coverage, medium-low coverage, and low coverage. The sample size was calculated for a broader study using structural equation models, in which several instruments are related, such as beliefs, knowledge, activity planning, and self-efficacy. Using an online calculator and the methodology described by Soper (2003) (45), for a small effect size of 0.1 (relationship between the instruments), a power of 80%, a number of 15 latent and 40 observed variables, and a level of reliability of 95%, it was estimated that at least 850 women needed to be interviewed. The sample size corresponding to 969 women also meets the requirement regarding instrument validation (46). The inclusion criteria were the characteristics of the universe previously. The exclusion criteria were the presence of CC and/or total hysterectomy. Recruitment was carried out by telephone or by home visit. The interviews were conducted by previously trained personnel.

The sociodemographic variables, adherence to the Pap test, and self-efficacy in relation to screening were measured during the interview. The self-efficacy variable was measured with the SES-PSSP questionnaire (Self-Efficacy Scale for Pap Smear Screening Participation) (47). This questionnaire, validated in the North American population, has 20 items distributed in two dimensions: the first, on personal costs, includes aspects such as time, money, transportation and interruptions of life; and the second, on relationships, which includes the opinion of family members and peers. According to the original recommendation of the author of the instrument, 2 items can be added in case the interviewed woman has children and can leave them alone; given that these items are not applicable to all women, the author of the instrument does not include them in the dimensions described above and therefore they were not included in this research either. The answers are measured on a 5-point Likert scale (1 corresponding to “I would definitely do the Pap test” and 5 corresponding to “I would definitely not do the Pap test”).

For the validation of the instrument, the translation and back-translation of the questionnaire was carried out by two professionals in their respective native languages (English and Spanish); it was later submitted for determination of validity of cultural content to five thematic experts. First, the original questionnaire was translated into Spanish by a bilingual (Spanish/English) and native Spanish professional and researcher, and the “Spanish version” was obtained. Second, a bilingual native English professional researcher translated the “Spanish version” into English. Third, another bilingual researcher compared the original and translated versions of the instrument to ensure that the meaning of each item was not altered. In this case, both versions matched; thus, no changes to the translated version were needed. In relation to content validity, the reviews by the five researchers—who analyzed the characteristics of each of the items in terms of their understanding and applicability to the context in which the instrument would be used—were considered. There were no suggested changes. Subsequently, the questionnaire was applied to 10 women from the population that would be studied, to find out if the questions were understandable and/or if there were any terms that prevented a fluid response; there were no suggested changes.

### Statistical analysis

The continuous variables were described using means and standard deviations, and categorical variables using absolute frequencies and percentages. Construct validity was performed using confirmatory factor analysis (CFA), criterion validity using Student’s t-test, and reliability using Cronbach’s alpha. Adherence to the Pap test was used as an external criterion of validity, for which the scores for each of the factors and the total score were calculated using the regression method. The scores of those who adhered to the Pap test were then compared with the scores of those who did not, using the t-Student test for independent samples. The regression method using a multiple ordinary least squares regression to predict each individual’s factor score based on their observed variables was used (48). The models were estimated using diagonally weighted least squares. The fit of the models was measured using the chi square statistic and two fit indices: the Comparative Fit Index (CFI) and the Tucker-Lewis index (TLI). The Root Mean Square Error of Approximation (RMSEA) was used as the parsimonious fit index. CFI and TLI values greater than 0.95, with RMSEA less than 0.05 are good; CFI and TLI values between 0.90 and 0.95, and RMSEA between 0.05 and 0.08 acceptable; and CFI and TLI values less than 0.90, or RMSEA greater than 0.08 unacceptable. The data were analyzed with the lavaan and psych packages of the R program. A p value <0.05 was considered significant.

The study was approved by the Ethics Committee of the Southeast Metropolitan Health Service, Santiago, Chile. Their signature of the informed consent document was requested from each of the women in the study.

## Results

The average age of the study group is 43.37 ± 10.77 years, and educational level is 10.97 ± 3.4 years. 63.7% of the women work for pay; 79.2% have a partner; 74.5% maintain sexual activity, with 2.69 ± 2.73 (range 1 to 40) being the number of sexual partners; 93.3% have children; and 58.9% use some method of family planning.

76.5% of the women (n = 741) reported having adhered to the Pap test in the last three years. Of the group of women who did not have a Pap test in that period (n = 228), 14% had never had a Pap test, and the remaining 86% reported having it for more than 3 years.

The items with their respective means and standard deviations are presented in **Table 1**.

**Table 1:** Means and standard deviations of items of the questionnaire.

How likely are you to get a Pap smear if:(¿Que tan probable es que usted se haga un Papanicolaou si):	Mean	Standard Deviation
1.1. How likely are you to get a Pap smear if your last Pap was normal?(¿Que tan probable es que usted se haga un Papanicolaou si Su último Papanicolaou fuese normal)?	1,58	0,932
1.2. How likely are you to get a Pap smear if you need a ride to your appointment?(¿Que tan probable es que usted se haga un Papanicolaou si alguien tuviera que llevarla a su cita para tomarse el Papanicolaou)?	1,88	1,174
1.3. How likely are you to get a Pap smear if you are too busy during clinic hours?(¿Que tan probable es que usted se haga un Papanicolaou si usted estuviera ocupada durante el horario de atención del consultorio)?	2,43	1,384
1.4. How likely are you to get a Pap smear if without applicable health insurance?(¿Que tan probable es que usted se haga un Papanicolaou si usted no tuviera seguro de salud para pagar el Papanicolaou)?	2,31	1,454
1.5. How likely are you to get a Pap smear if someone in your family tells you the Pap is unnecessary?(¿Que tan probable es que usted se haga un Papanicolaou si alguien en su familia le dijera que el Papanicolaou no es necesario)?	1,59	1,035
1.6. How likely are you to get a Pap smear if it is hard to get a provider to take your insurance?(¿Que tan probable es que usted se haga un Papanicolaou si usted tuviera problemas para encontrar un médico o matrón (a) que atienda con su seguro de salud)?	2,11	1,327
1.7. How likely are you to get a Pap smear if you have a frequent change of residence?(¿Que tan probable es que usted se haga un Papanicolaou si usted se cambiara de casa frecuentemente)?	1,92	1,236
1.8. How likely are you to get a Pap smear if a close male friend or your husband tells you a Pap is not needed?(¿Que tan probable es que usted se haga un Papanicolaou si un amigo cercano o su pareja/marido le dijera que el Papanicolaou no es necesario)?	1,51	0,960
1.9. How likely are you to get a Pap smear if you have irregular vaginal bleeding?(¿Que tan probable es que usted se haga un Papanicolaou si usted tuviera sangramiento vaginal irregular)?	1,30	0,692
1.10. How likely are you to get a Pap smear if without permanent housing?(¿Que tan probable es que usted se haga un Papanicolaou si usted no tuviera un lugar donde vivir de manera permanente)?	1,98	1,292
1.11. How likely are you to get a Pap smear if friend(s) tells you a Pap is unnecessary?(¿Que tan probable es que usted se haga un Papanicolaou si su amiga(s) le dijera que el Papanicolaou no es necesario)?	1,50	0,946
1.12. How likely are you to get a Pap smear if your Pap is self-pay?(¿Que tan probable es que usted se haga un Papanicolaou si usted tuviera que pagar por el Papanicolaou)?	1,84	1,242
1.13. How likely are you to get a Pap smear if you are drinking alcohol heavily?(¿Que tan probable es que usted se haga un Papanicolaou si usted bebiera mucho alcohol)?	2,36	1,490
1.14. How likely are you to get a Pap smear if you would lose work time?(¿Que tan probable es que usted se haga un Papanicolaou si usted tuviera que faltar al trabajo)?	2,05	1,375
1.15. How likely are you to get a Pap smear if you are living in a drug treatment place?(¿Que tan probable es que usted se haga un Papanicolaou si usted estuviera en un centro de rehabilitación por drogas)?	2,39	1,481
1.16. How likely are you to get a Pap smear if without a regular health care provider?(¿Que tan probable es que usted se haga un Papanicolaou si usted no tuviera un profesional de salud que la atienda regularmente)?	2,01	1,298
1.17. How likely are you to get a Pap smear if on street drugs?(¿Que tan probable es que usted se haga un Papanicolaou si usted estuviese usando drogas)?	2,63	1,552
1.18. How likely are you to get a Pap smear if you had a past abnormal Pap?(¿Que tan probable es que usted se haga un Papanicolaou si usted hubiese tenido un Papanicolaou alterado/anormal en el pasado)?	1,17	0,576

The Spanish version is shown in parentheses.

**Figure 1:**
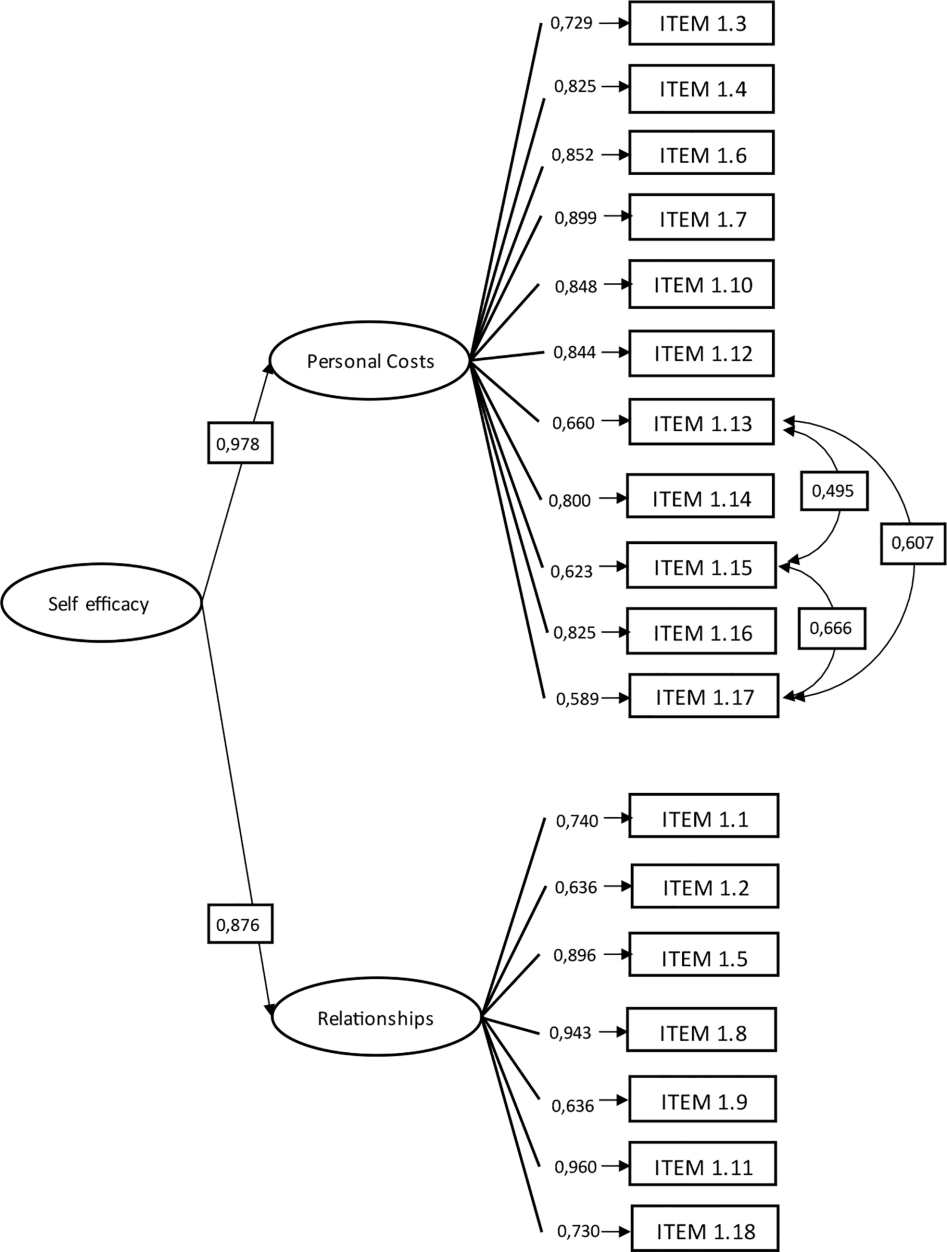
Factor loadings of first and second-order and correlations between items of the final model (n = 969).

For construct validity, the first model tested considered the distribution of the 20 items in the two factors of the original instrument. Given that the fit indices were not good, and the modification indices suggest transferring item 1.3 to the personal costs dimension, a second model was tested. The change of the item is welcome since the meaning of this corresponds to a personal cost. The second model showed acceptable adjustment indices; however, a correlation of 0.857 between both factors was presented, which suggested testing a second-order model. The third model tested was second-order; the results indicated acceptable adjustment indices, so it was decided to retain it. The fit indices of the three models tested are presented in **Table 2**. The standardized parameters of the final model and the significant correlations between the items are presented in **Figure 1**. Cronbach’s alpha for the total instrument is 0.95, 0.94 for the personal costs dimension, and 0.91 for the relationships dimension. The results of criterion validity are presented in **Table 3**.

**Table 2:** Fit indices in the three tested models of the SES-PSSP questionnaire (n = 969).

Models	χ^2^	gl	p value	CFI	TLI	RMSEA(CI 95%)
First (Original questionnaire)	1831,976	134	<0,001	0,952	0,945	0,114 (0,110–0,119)
Second (Change ítem 1.3)	592,374	131	<0,001	0,987	0,985	0,060 (0,055–0,065)
Third (Second-order)	591,810	131	<0.001	0,987	0,985	0,060 (0,055–0,065)

**Table 3:** Criterion validity by comparing groups according to adherence to the Pap test.

	Pap test in the last three years		
	Yes	No	
Factor	M ± DE^†^	M ± DE^†^	p value (^*^)
1. Personal Costs	0,461 ± 0,722	−0,139 ± 0,753	<0,001
2. Relationships	0,495 ± 0,802	−0,128 ± 0,809	<0,001
Total score	−0,448 ± 0,694	−0.133 ± 0,725	<0,001

^†^M ± DS: Media ± Standard deviation ^(*)^ Student’s t-test was used to compare groups.

The standardized scores for each of the factors do not have an absolute meaning but a relative one. Being significantly different, it is to be expected that some will be positive and others negative.

## Discussion

The reduction of mortality and morbidity due to CC requires, among other things, the identification of factors that allow predicting adherence to Pap test; self-efficacy is a construct that had been related to CC screening. The main contribution of this study is the validation of an instrument to measure self-efficacy for taking Pap test, which can be very useful in both health care and research. Although there is another instrument validated in the Latino population that measures self-efficacy on this same topic (49), the SES-PSSP is important since it measures different situations that women could hypothetically face when deciding whether to adhere to screening. The possibility of posing different situations is a necessary condition to efficiently measure self-efficacy (2).

CC is an important public health problem in Latin America and the Caribbean. Therefore, having a questionnaire in Spanish will be very useful in measuring the self-efficacy of women and developing interventions to increase it because enhancement programs result in increased screening rates (50–52). Findings suggest that the inclusion of self-efficacy information in entertainment programming may lead to beneficial health outcomes (35).

Although the questionnaire was validated in the Chilean population, its usefulness transcends borders, since the Latino population shares cultural values that explain many health behaviors, including barriers to adherence to CC screening (53). Latina women in the United States have greater CC mortality rates than non-Latina women because of their low rates of screening (54). Receiving provider advice both directly and indirectly predicted Pap test adherence through greater self-efficacy (55). A systematic review found that self-efficacy is also a facilitator to CC screening in young people (56).

Related to the construct validation, in general, the factor loadings of the CFA are higher than in the PCA. This is a consequence of having a second-order factorial analysis, with different loads for each one of the dimensions, and therefore, the role of the items in each of the dimensions appears a little more precise.

The CFA carried out using a second-order model supports the two original dimensions proposed by the author of the SES-PSSP, and therefore provides sufficient evidence to consider the instrument valid and reliable. The confirmatory analysis in the Chilean population provides new evidence that both factors, validated in the original instrument, are explained by a second-order factor, self-efficacy. Although there is a difference between the characteristics of the women in the validation of the original instrument (47) and the Chilean sample, the instrument was maintained with the same items with high factor loads.

The change in item 1.3 from the relationship dimension to the personal cost dimension may be explained by the differences that exist between the women in both studies. The North American sample is an institutionalized population (inpatient), while the Chilean sample was drawn from the population belonging to primary health centers. Therefore, the fact of “being busy during office hours” is a personal cost for the Chilean woman, while for the North American, she is dependent on others. It has been previously described in the Chilean population that both office hours and waiting time are a difficulty for women when deciding to adhere to screening (57). The context in which each woman finds herself determines this difference.

When analyzing the moderate correlations between the items that are not explained by belonging to the personal costs factor, these could be explained by the three items referring to the use of alcohol or drugs. Since none of the correlations presented values above 0.8, all the items were kept in the instrument.

Finally, the results of the criterion validity provide additional strength to the instrument since higher scores in the total and in both dimensions of the instrument are significantly associated with adherence to the Pap test.

## Conclusions

The World Health Organization’s efforts to eliminate CC by 2030 with a target of 70% screening coverage using a high-performance test necessitate that women increase participation in screening (58). Self-efficacy is a construct that has proven to be very useful in explaining health behaviors, and specifically to be included in interventions aimed at increasing women’s adherence to CC screening. Therefore, having a validated and reliable instrument in the Spanish language is very useful, both for professionals in the clinical field and those who carry out research in the area.

## Data availability statement

The raw data supporting the conclusions of this article will be made available by the authors, without undue reservation.

## Ethics statement

The studies involving human participants were reviewed and approved by Ethics Committee of the Southeast Metropolitan Health Service, Santiago-Chile. The patients/participants provided their written informed consent to participate in this study.

## Author contributions

MT-U and OP contributed to conception and design of the study. MT-U and OP organized the database. OP performed the statistical analysis. MT-U wrote the first draft of the manuscript. Both authors contributed to manuscript revision, read, and approved the submitted version.

## Funding

FONDECYT #11130626 “Determinantes Sociales para la Adherencia al Tamizaje de Cáncer Cervicouterino”/Social determinants for adherence to Cervical Cancer screening”

## Conflict of interest

The authors declare that the research was conducted in the absence of any commercial or financial relationships that could be construed as a potential conflict of interest.

## Publisher’s note

All claims expressed in this article are solely those of the authors and do not necessarily represent those of their affiliated organizations, or those of the publisher, the editors and the reviewers. Any product that may be evaluated in this article, or claim that may be made by its manufacturer, is not guaranteed or endorsed by the publisher.
